# Challenging the Hidden Curriculum through Problem Based Learning: A reflection on Curricular Design

**DOI:** 10.15694/mep.2018.0000159.1

**Published:** 2018-08-06

**Authors:** Lorena Hough, Iman Hegazi

**Affiliations:** 1 Western Sydney University

**Keywords:** Problem-based learning, patient-centered care, hidden curriculum, narrative, emotive language

## Abstract

This article was migrated. The article was marked as recommended.

Problem-Based Learning pedagogy has been around since the late 1960s. However, PBL case-writing still constitutes an arduous task with much debate about how the cases should be written. The influence of the different PBL-writing styles and approaches have upon medical students requires attention and research. The Western Sydney University medical school has recently undergone a PBL renewal project, redesigning and rewriting 52 high quality PBL cases. Much thought and consideration were exerted in the planning and delivery of this project with a focus not only on the deep-learning and understanding of the basic and clinical sciences, but also on the importance of patient-centeredness. In this paper, we emphasize how language used in PBL cases can have a profound impact on students through implicit learning and the hidden curriculum. We also recommend having a writer, experienced in the use of narrative and emotive language, in addition to clinicians and medical educators in the authorship of PBL cases.

## Introduction

Problem-Based Learning, as a pedagogical approach, has been around now for more than half a century ( Neufeld and Barrows, 1974). However, the way PBL cases are written and presented still differs wildly across different universities and sectors. The influence these different styles and approaches have upon students has not been widely studied ( Kenny and Beagan, 2004, MacLeod, 2011).

The Western Sydney University medical school has recently undergone a renewal project, rewriting 52 PBL cases which span the preclinical years of its undergraduate program. In planning and implementing this renewal, we thoughtfully considered our approach, realising that our focus should be not only on deep-learning of the basic and clinical sciences, but also that each case should be strongly patient-centred. In this paper, we will examine what a patient-centred PBL case means, its relation to the hidden curriculum, and the method by which we crafted each case.

## What is a patient-centered PBL case?

The term ‘patient-centred’ is in common usage in medical practice, referring in general to healthcare in which doctor and patient work together, and where the patient is able to make informed choices about their care ( Rawson and Moretz, 2016). However, it is important to look deeper than that, particularly in medical education. Patient-centredness is ideally a mindset, a way of thinking; as opposed to a set of rules. The human experience is messy and complex, and a patient-centred doctor will see the sick person in this way rather than simply as a disease host. According to Epstein, “behaviours associated with patient-centred care, such as respecting patients’ preferences, should be justified on moral grounds alone, independent of their relationship to health outcomes” ( Epstein and Street, 2011). Medical teachers have the responsibility to assist students in developing this mindset for their growth as healthcare professionals.

PBL is an ideal platform for students to explore the patient-centred mindset. Crucially, it can also be a platform in which students first learn to dismiss the patient as a person and to focus only on the disease. PBL is in this sense a power for good or evil, depending on how the case is written and how it is taught ( Kenny and Beagan, 2004). Language is rarely wholly objective and when it is used in a narrative, however plain, short or simple it may be, it will always carry a wealth of subtext. Its meaning and interpretation go much deeper and spread much wider than the literal meaning of the words. For this reason, a key contributor to the writing of a patient-centred PBL case should be someone with narrative expertise. The patient should sit at the heart of this narrative; in other words the doctor is not the ‘hero’. In general, we aimed to have the doctor in each case provide the function of a competent figure who facilitates the patient’s journey through illness and recovery.

Therefore, the use of narrative in patient-centred teaching requires a purposeful, informed method of delivering a subtext which is genuinely patient-centred. McLeod, outlines six ways PBL cases can sabotage student learning, and all six ways deal with the subtext of narrative. ( MacLeod, 2011) Positive ways to construct the narrative will be explored later in this paper.

## PBL and the hidden curriculum

The concept of the hidden curriculum in medical education has been well explored in the literature, though it is often focused on clinical encounters ( Hafferty F W and O’Donnell, 2014). The hidden curriculum deals with the unspoken aspects of teaching and learning; of the acquisition of attitudes, perceptions, culture and subculture within the healthcare profession and the medical school ( Dent and Harden, 2013). Naturally, the focus is usually on person-to-person interactions between doctor and student, and between doctor and patient. However, for many medical students, one of their first encounters of the hidden curriculum’s impact comes with PBL. From the initial presentation, the hidden messages are automatically present.

Consider the most generic format of initial presentation in a PBL case, such as:

*Janet Smith, age 38, presents with dyspnoea and right-sided chest pain to a GP clinic. She reports that the symptoms commenced approximately 24 hours ago.*

What hidden messages are present here? The most evident is that science and symptoms are the serious subjects at hand: the fact that they are attached to a person is irrelevant. Other hidden messages reveal that an ideal doctor-patient initial encounter is straightforward and factual, and there is perhaps, arguably, just the slightest hint of disapproval towards a patient who has not reported her symptoms until 24 hours have elapsed.

Naturally, a PBL case cannot read like a novel. It is not the place to spend pages of writing analysing the patient’s every mood and reaction or the backstory of her entire life. Emotive language must be used sparingly, or it risks alienating students through an obvious attempt to manipulate their feelings. Writing an effective PBL can, in terms of language, mean treading a fine line between a patient appearing as a cardboard puppet, or an actor in a melodrama. However it is not as difficult as this may sound. The key is, in every stage of PBL writing, simply to keep forefront in the writer’s mind that the patient is a human being; a person who simply *is*, in their given situation (rather than someone to be defined, categorised and judged).

## Constructing and writing the PBL case: The patient’s name

McLeod’s is an excellent study on the way patient names can damage student learning. Some of the patient names she lists, which have actually been used, are quite shocking. ( MacLeod, 2011) She notes that in a given year at one university, PBL doctor names were prestigious (e.g. J.F. Kennedy) while patient names were denigrating (e.g. Roger Suicide-Wish or Suzie Fusspot). Worse, the denigrating names tended to be assigned to low socio-economic patients, and to patients who are otherwise stigmatised such as those with obesity, anxiety or alcohol dependency. There is certainly a place for humour in medicine, but it surely should be self-evident that using humour to glorify doctors and mock the sick and underprivileged is unacceptable in any situation. To do so in a PBL is a very strong example of negative hidden curriculum, as actually embedded in the “formal” curriculum. This amplifies the conflict that already exists between values adopted in other areas of the formal curriculum and those observed within the hidden curriculum, causing cynicism and confusion among medical students and imbuing them with a sense of powerlessness, especially in the context of addressing the conflict ( White, Kumagai et al. 2009).

The example given above of ‘Janet Smith’ is obviously a generic name. In reviewing our old curriculum, many of the names were similarly generic. In one case the patient’s name actually morphed from William Smith to Tom Smith, and it is telling that this went largely unnoticed. A generic name gives the hidden message to students that this patient is simply a representation, not a person: forgettable, unrealistic and unimportant.

When writing our new PBL cases, we placed real importance upon choosing the patients’ and doctors’ names. A person’s name can tell a great deal about them: their gender and race, something of their generation, perhaps a clue to their parents and upbringing. Our university is located in, and serves, Greater Western Sydney (GWS), and we wrote our cases to reflect the demographic of the area. GWS is a diverse, multicultural area with a predominantly high percentage of low socio-economic areas. The 2016 census showed that 33% of GWS population came from countries in which English was not their first language and that 18% of the population migrated from overseas within the previous five years. The census data also showed a 7.1% unemployment rate, and 11.6% of households with a weekly income of AU$2,000 - $2,499. ( Census, 2016)

We looked for patient names which had something to say, and which also felt realistic rather than fanciful. Thus we chose names such as: Jayden Harrison (age 3); Margery Hodge (age 81); Sanjana Joshi and Adrian Zhang. Adrian Zhang’s name indicates not only his Chinese heritage, but also the probability of his being born in an English-speaking country, perhaps second or third generation Australian. In contrast, we named another patient Ling Hai, an older man who migrated to Australia in his twenties and whose family name ‘Ling’ is put first, in the Chinese way. Throughout the case he is then referred to as Mr Ling, although in general we decided to refer to most patients by their first names. This decision was based on common and recommended practice ( Davies-House, Ball et al. 2017).

Doctors’ names involved less thought, but we aimed to give variety of race and gender. Thus, we used names which are ordinary but not generic, e.g. Dr Linda Fitch, Dr Muhammad Khan and Dr Chowdhury. We also used the second person for a number of PBLs. Some cases begin, “You are a medical student observing the practice of Dr…” and other cases the “you” would in fact be the doctor. Still other cases were written entirely in the third person.

One difficulty of using the second person is when the patient is referred to a number of different health professionals. It is unrealistic that a medical student or GP can follow the patient everywhere they go. Sometimes this factor determined whether to write a case in third person, as when the patient is moved around to many places; but in other cases we allowed the “you” character to quietly fade away while the patient moved on. However, as long as the “you” character is present as a medical student or intern, we tried to ensure they remained realistically present, e.g. the GP might ask the patient’s permission for the medical student to observe the consultation, or the “you” character may observe that the GP is having difficulty communicating with the patient.

## The patient’s demographic

In our aim to represent the demographic of Greater Western Sydney in our PBL patients, we particularly needed to consider the relationship between representation and stereotyping. For example, we had to consider how to present the HIV patient. The original case we were replacing used a doctor with a needlestick injury, which is currently a very rare occurrence. In having to make it a sexually transmitted infection, we were faced with the difficulty of stereotyping homosexual men. The content expert clinician we consulted explicitly stated that the only realistic portrayal of a patient with HIV would be a homosexual man, of which he said at least 75% of his patients are. But it was important to consider more than mere representational statistics. We were concerned that making our HIV patient homosexual might a) offend, alienate or humiliate homosexual students in the classroom; b) strengthen a pre-existing stereotype of gay men as promiscuous and disease-ridden; and c) prevent medical students from thinking more widely about the ‘type’ of people who might contract HIV and/or the conditions suffered by homosexual men in general.

In attempting to address this issue realistically, but without stereotyping, we eventually decided to leave our patient’s past sexual history a little unclear. He was married to a woman for about five years before the marriage broke up; since then he has had no sexual partners. Further history taking reveals that he backpacked extensively in his early twenties in various parts of the world and in his words, “may have had some adventures” there.  Whether or not his adventures were heterosexual or homosexual, or whether it was drug-sharing with the same needle, is left unclear, as the patient has no desire to give any details. The PBL case does cover the necessity of contact tracing, although in this case it is impossible to contact any of our patient’s sexual partners apart from his ex-wife. This method of conducting the scenario, we hope, gives a positive hidden curriculum message: that a) the patient has a right to keep some information back, as the diagnosis remains the same regardless of its cause; b) the doctor does not demand private information from patients unless it is necessary for medical treatment and/or the safety of others; and c) medical students are more free to think about all the options and possible causes for HIV in this patient without being confined to one obvious cause. We also added the true demographic statistics as one of the questions to be discussed in class, as realistic statistical representation is still important for students to cover. Additionally, in another PBL case about thrombosis and warfarin, we incidentally represented this patient as homosexual: a middle-aged Japanese businessman who refers to his partner Mark. No further investigation is made into this patient’s sexual history as it is not relevant to his condition.

We considered this theme of representational *versus* stereotypical in many other patient contexts as well. For example, we needed to ensure that not all Aboriginal patients would be represented as low socio-economic. Thus, although we did have one woman on carer’s benefits, there is another case in which the patient is a HR officer for the local government council. We also did a longitudinal case following up the woman on carer’s benefits. This patient first appears in a first-year Type 2 diabetes case (which in itself we worried might be strengthening a stereotype) and followed the same patient up in a second-year renal case. Here our patient’s life had moved on, fifteen years later, she has completed a college degree and is enjoying her new career in a legal office.

We tried to avoid other common stereotypes, for example the patient with alcohol dependence is a lecturer in modern history and a poet, replacing the old case which depicted an unemployed, heavily tattooed man. On a smaller scale, we made sure to create a mix of family situations (single parents, marriage, de facto etc.), and of course a range of cultural and socio-economic backgrounds. For all of these “types”, we tried to make the patients as well-rounded as possible. Sometimes they did fit into a general stereotype to some extent. The case about obesity focuses on a lower middle class white family in an unremarkable suburb in the outer southwest of Sydney. All members of the family are overweight to obese. This, if not a stereotype, is surely an archetype of ordinary living in outer suburbia, as well as depicting a demographic commonly associated with obesity. In writing this case, we used a lot of dialogue and character development, in an effort to make our family not less ordinary, but more like real people. Real people, of course, are always complicated and although PBL is not a place to develop enormous detail, it can allow for depiction of personality. The following quote is from the history section, which hopefully illustrates the broad brush strokes which try to paint a very ordinary family of individuals.

*You also ask Karen about the other aches and pains the family suffers. She sighs. “Well, it’s hard to pin them down,” she says. “I get a sore neck sometimes, and my husband Steve gets a bad back that comes and goes. Caitlin, who’s in Year 6, often has a tummy ache. Jacob, my 15-year-old son, doesn’t complain of anything but he never seems to be happy. He hides out in his room most of the time. That’s probably normal for a teenager, but I don’t know – I’m worried he might be having a hard time at school.”*

It is perhaps worth noting that Jacob presented difficulties to us as writers. There was not room enough in this PBL case to explore his issues, whether stemming from bullying, depression, other mental health problems, or if it is indeed normal adolescent behaviour. We tried to negotiate this by a sort of cliff-hanger at the end:

*You become increasingly concerned about Jacob, whose reclusive habits are becoming more marked. Karen is worried and distressed about him and can’t get him to talk to her about it. You ask if Jacob might come in and see you for a future appointment.*

Stereotypes are reinforced by many small factors. In the cases we were replacing, we found that not only the patients’ names were generic, but their jobs, too. The vast majority of adult patients in the old cases were teachers, students, or health professionals. Exceptions tended to be either directly related to the condition (sleep apnoea patient was a truck driver) or to the patient’s racial background (Vietnamese immigrant worked in a clothing factory, Turkish immigrant was a fruit picker). In writing our new cases, one of our enjoyable tasks was thinking up a wider range of employment for our patients. This makes sense as our students will graduate and work in several hospitals and meet patients from all sorts of backgrounds. There is currently a proposal that higher education should embrace general/life education to help equip students for life as well as work in the 21st century ( Bussemaker, 2016), and in the case of medicine and similar professions work and life experience become heavily interwoven. Many of our PBL patients’ professions are more or less ordinary: a bookshop owner, a phone technician, a taxi driver. Some were a lot more unusual, though we were careful to keep these to a reasonable minimum: a male ballet dancer, a Ghanaian astronomer visiting Sydney for a conference, a High Court barrister.

Looking back, for all our focus on demographics, there are still things we could improve upon and add. In particular, we could have created some of the patients as people with a disability unrelated to the condition being studied. This would ideally mean one patient have a mental disability and another have a physical disability. We also should have included a female homosexual patient.

In addition to simply creating these demographic differences in our patients, we also made sure this remained anchored in the patient’s journey through healthcare – that it was not immediately forgotten about in the pursuance of diagnosis and treatment. For example, a patient on a remote farm station has one of her GP appointments via Skype. A low socioeconomic patient explores options for support with paying for medications and healthier food. A fit young woman diagnosed with coeliac disease rejects the offer of a dietician, choosing instead to do the research herself – the pros and cons of this are mentioned within the PBL case.

## The patient’s photograph

Most PBL cases start with a photograph of the ‘patient’. This image can do a lot to influence the hidden messages. Again, generic issues still apply. If the photo itself is too small, pixelated, or obviously taken from stock images or a website somewhere, this immediately detracts any amount of good which may have been done in the story itself, portraying the message of ‘quick fix’ and that ‘this is not reality’. Photographs need to be of high image quality and to not be immediately recognisable as a ‘type’, such as is used in marketing stock images.

The first choice with the patient’s photograph is whether to depict the patient in their current state of illness – e.g. being wheeled in a stretcher, or in the act of vomiting. This, if done well, can be effective; however if it looks fake it can undo all good done in the case, and break the feeling of reality. If students are amused by the photo, it’s a good sign it needs replacing.

If the choice is made to use a straightforward portrait shot of the patient, there are still many decisions to consider. For example, if the patient is a male construction worker, his photo should not look like an aftershave advertisement. On the other hand, we would also need to be wary of using a stock photo image of a brightly smiling (or seriously posing with arms folded) man in a high-visibility vest and an orange helmet, as this will appear equally contrived. A glossy brochure effect will detract from the feeling of reality just as much as a poor-quality image.

Our approach to the portrait photos was to aim for a casual snapshot appearance, as they might look if one met them on the street or in the doctor’s waiting room. We bought some stock images, but more often we made use of section 49 of the Copyright Act 1968, and used high quality, representational photos from news articles online and from Wikimedia Commons, carefully vetting each one to ensure it did not look too posed or ‘glossy’. Some photos we took of people we knew; but this proved increasingly difficult as finding someone of exactly the right age and cultural background who is willing to pose was harder than we first imagined it would be, particularly since using staff members was too difficult as the students would know them.

## Language

Words and language used within a PBL case can make or break the positive impact of the hidden curriculum. Some of the words and phrases in common usage within medicine are enormously loaded with hidden judgment, blame or disregard.

Commonly in receiving notes from clinicians for use in PBL cases, the word “complains” appears: “Ms Freeman complains of lower back pain,” for example. If this same word is translated to a patient scenario, there is a flavour of weakness and peevishness in the word as applies to the patient. If using formal language, it is much more objective to say “Ms Freeman reports lower back pain,” and when taking a narrative approach, using more words, e.g. “Tina explains that she has a constant ache in the lower part of her back, and that the only way to ease it is to lie on the floor.”

Another word we were careful of is “compliance”. If a patient is labelled ‘non-compliant’, or even ‘non adherent’, it implies immediate judgment. These are paternalistic terms, with an underlying assumption that the patient should obediently follow instructions without needing to agree or understand why ( Randall and Neubeck, 2016). In combating this problem in PBL, we aimed to not use any generic term at all to explain. We would instead provide a narrative which involved the medication not being taken, e.g.

*“For the first few years after her diagnosis of Type 2 diabetes, Alinta regularly took her medications and kept up her diet changes. But since Aunty Beryl died and the children moved out, Alinta has felt like a new person and old routines and habits faded out. Alinta never made the decision to stop, but she felt so well and so interested in her new life that she just kept forgetting until it slipped off the radar altogether. On her last visit to Dr Patten five years ago, she renewed her medications again but it soon faded out once more”.*

It could perhaps be argued with reason that this is still judgmental, and has the appearance of trying too hard to make excuses for the patient. However, it does give a much clearer picture of the patient as a sentient being with a mind and life of her own, rather than the judgment-laden bald statement, “Alinta has been non-compliant with her medications for approximately seven years”.

## Using the patient’s voice

In writing the new cases, we made a point of including a direct quote from the patient in the initial presentation of every case. Often, we would also use the patient’s direct voice in other parts of the case as well. This does several things. Firstly, it gives the patient a voice in the political sense of the word – the patient is directly involved in the process from the very beginning. Secondly, it makes the patient more present, more real, and can give a sense of the patient’s personality by the words and phrases used. Thirdly, it means the presenting symptoms are conveyed in non-technical language, as in the real world, providing more room for interpreting and exploring, as well as bringing the patient’s symptoms to life.

Thus in the opening presentation of the Multiple Sclerosis case the patient, a professional ballet dancer, is given a voice as follows:

*“It’s pretty worrying,” Aaron says. “I fell in rehearsal the day before yesterday. I can’t understand it, it was just like my leg gave way beneath me. And since then that same leg, it’s my left, keeps feeling as if it might give way again. I definitely need to have it checked out.”*

The following History section of the PBL is mostly told without dialogue; however his voice is used again briefly to clarify details of his fall:

*“I don’t blame my technique – it was a basic brisé, the kind of thing I could do in my sleep,” he says. “It just happened.”*

We wanted the use of direct dialogue in these cases to serve multiple functions at once, never to be just used as scene-setting padding. The above quotes are giving definite information relating to his presenting symptoms; however they are couched in the kind of language a lay-person would use. The benefits of using the patient’s voice is lost if their words are simply parroting what would have been written without the quotation marks, e.g. (to give a slightly exaggerated example) “I have noticed episodes of lower limb weakness recently on my left side.” As writers we imagined ourselves as the patient, and said the kind of things the patient, as a real person, might say.

The patient’s voice can also be used to give information that would seem contrived otherwise. For example, in a PBL case about Graves’ Disease, the patient visits her GP for a check-up of her six-month-old son. After the check-up, the GP asks how she, the mother, is going. The patient replies in quotes, that she’s been tired and stressed, sleeping badly and adds, “That’s motherhood for you!” The GP (her long-standing family doctor) then comments on how the patient looks as if she might have lost weight.

*Angelica laughs. “Yeah – I’ve lost my baby weight and then some.” She looks down at Noah. “See what you’re doing to me, you little monster you! You’re better than a gym, you are!”*

Without the dialogue, the information given might have sounded more contrived, e.g. “Angelica reports that her sleep patterns are disturbed and that she has lost 2.5 kg since her weight before pregnancy.” Angelica would not have realistically visited the GP on purpose to report this, as the symptoms are easily consistent with caring for a baby. Of course, the incidental nature of this information could still have been portrayed without the direct quotes, but using her voice adds a sense of how unconcerned she is about her own symptoms, giving a sense of the ordinary, if chaotic, daily life of a young mother.

In giving the examples above it may appear that our PBL cases are dialogue-heavy, but this is mostly not the case. In general the initial presentation scene, normally about half a page long or less, is dominated by dialogue, but after that the narrative is more a relation of interrelated fact and story.

## Take Home Messages

Language can have a profound impact on the hidden curriculum in medical education. Connotations of judgment, apathy, dismissal, or inappropriate humour can inadvertently reveal and enforce attitudes educators are trying to combat. In Problem-based learning cases, not only language but also the way the patient is depicted can increase or destroy the positive attitudes taught in the formal curriculum. It is beneficial to include, in the PBL-writing team, a person with experience in the use of emotive language, and the ability to understand and wisely use the power of suggestion and connotation in PBL writing to positive effect.

## Notes On Contributors

Lorena Hough is a professional writer and a former employee of Western Sydney University, School of Medicine. Lorena played an important part in the authorship of fifty-two high quality PBL cases, and their tutor manuals, which were written over three years.

Iman Hegazi is the Director of Medical Education and PBL coordinator at Western Sydney University, School of Medicine. Iman led the PBL case-writing project and played an important role in coordinating the writing team, and in the writing, review and evaluation of the PBL cases.
