# Supplementary material for: Challenging the Hidden Curriculum through Problem Based Learning: A reflection on Curricular Design
Source: MedEdPublish (2016). 2018 Aug 6;7:159. [Version 1] doi: 10.15694/mep.2018.0000159.1 (PMC10701804; doi:10.15694/mep.2018.0000159.1)
Supplement: Peer review history

# Reviewer report: Reviewer response for version 1

**DOI:** 10.21956/mep.19634.r29243

**Published:** 2018-08-17

**Authors:** Faraz Khurshid

**Affiliations:** 1 University of Buraimi, College of Health Sciences

This review has been migrated. The reviewer awarded 5 stars out of 5 'Educative & Refreshing'This paper can surely serve as an important 'guideline' for all the professional and non-professional PBL writers and narrators. Still, it can provide a potential team collaboration between PBL writer and storyboard (case) designer to pre-visualise the case narration in a sequence with progressive disclosure. The patient-centric approach should always lie at the heart of PBL scenarios. The authors brought an insightful piece of information that can help to significantly improve the 'face-value' of PBL cases, such as deciding the name of the patients and doctors, emphasizing the demographic context of the scenario and enclosing the patient photographs. As the authors mentioned that PBL is usually the first confrontation of students towards hidden curriculum, revamping the PBL writeup guidelines (as suggested by the authors) can help to transform the strongly negative hidden curricular aspects the PBL with a positive hidden curriculum. Furthermore, the overall learning experience will be eventually complemented by the apparent formal curriculum. The high quality of the PBL scenarios will surely make a difference in teaching, learning and assessment.My humble gratitude to both the authors for sharing this ‘Food for thought’.

Reviewer Expertise:

NA

No decision status is available

# Reviewer report: Reviewer response for version 1

**DOI:** 10.21956/mep.19634.r29245

**Published:** 2018-08-17

**Authors:** David Taylor

**Affiliations:** 1 Gulf Medical University, Ajman, UAE

This review has been migrated. The reviewer awarded 5 stars out of 5 This paper is important, and worth reading, because it describes, with real insight, those lessons which many of us have learnt slowly and painfully! Making sure that the cases appear as real as possible is crucial, and the authors describe ways in which this can be achieved. There is scope for evaluation, of course, and the biggest difficulty there will be in accounting for the variety in skills of the facilitators. I felt that the sections on language and names were particularly valuable, especially in the light of inculcating a sense of the importance of the individuality of the patients (rather than simply using them as a vehicle to ensure the meeting of indicative learning objectives).Several places are now moving in the direction of virtual patients (VPL, for instance), but the lessons learned from this study transcend the medium and the highly skilled people writing and designing the virtual patient could usefully read and digest the material in this paper.

Reviewer Expertise:

NA

No decision status is available

# Reviewer report: Reviewer response for version 1

**DOI:** 10.21956/mep.19634.r29244

**Published:** 2018-08-12

**Authors:** Trevor Gibbs

**Affiliations:** 1 AMEE

This review has been migrated. The reviewer awarded 5 stars out of 5 I was drawn to reading and reviewing this paper simply and initially because of the conflict that the title raised in my first reading- the hidden curriculum and PBL. To me , BL is about writing a scenario that leads to the exploration of issues that lead the student to towards the expected learning outcomes. If we know those learning outcomes then they cannot be part of the hidden curriculum. I was pleasantly surprised however to find that it was more about patient-centredness and making sure that the "real" was introduced into the PBL cases and that the students explored all three domains of learning. The new approach to writing the scenarios appears very exciting and a longer term evaluation of how these new scenarios affect student learning and assessment is to be encouraged. A paper that all concerned with PBL and the writing of the scenarios should read.

Reviewer Expertise:

NA

No decision status is available

# Reviewer report: Reviewer response for version 1

**DOI:** 10.21956/mep.19634.r29242

**Published:** 2018-08-07

**Authors:** David Bruce

**Affiliations:** 1 None

This review has been migrated. The reviewer awarded 4 stars out of 5 In this thoughtful paper the authors describe how they have renewed their medical school PBL cases giving a patient centred focus. The methodology used and their emphasis on the importance of language and writing skills is set out. The crude patient caricatures of past years such as “Suzie Fusspot” and the impact this has on the hidden curriculum certainly brings home the need for continual review of PBL material. In their careful consideration of patient names, demographics and photographs it seems to me that the PBL patients are made more authentic for the students. The authors avoid judgement laden phrases in the patient presentations such as the “complains of” and this is changed to “reports”. However, while patient rarely complain about anything, they also do more than report – they are concerned, anxious, curious, bothered etc – and adding this may enhance the authenticity of the scripts. This is achieved par excellence by the use of the patient’s voice in the narrative – a hint at the reasons and concerns for this presentation. The purpose of the PBL case must be to stimulate the students to explore and learn the medicine behind the presented problems. I think the authenticity of the patients used in the cases and hearing their narrative does give a patient centred focus. A PBL scenario is not set up to explore why this patient presented today with this problem or what this problem means for this unique patient, but in their careful renewal of the narrative the authors aim to have students explore and learn medicine with a patient centred focus.The renewal project described is well thought through and evidence based. Given the considerable work that has gone into this I do feel some follow up and evaluation is needed - will it help students become patient centred doctors? As one might expect – a well written paper!

Reviewer Expertise:

NA

No decision status is available
